# Epigenetic aging of human blood cells is influenced by the age of the host body

**DOI:** 10.1111/acel.14112

**Published:** 2024-03-04

**Authors:** Petter Holland, Mette Istre, Maryan M. Ali, Tobias Gedde‐Dahl, Jochen Buechner, Mari Wildhagen, Sonja H. Brunvoll, Steve Horvath, Shigemi Matsuyama, John Arne Dahl, Friedrich Stölzel, Arne Søraas

**Affiliations:** ^1^ Department of Microbiology Oslo University Hospital Oslo Norway; ^2^ Department of Internal medisin Bærum Hospital Drammen Norway; ^3^ Department of Hematology Oslo University Hospital Oslo Norway; ^4^ Department of Pediatric Hematology and Oncology Oslo University Hospital Oslo Norway; ^5^ Altos Labs Cambridge UK; ^6^ Department of Medicine, Department of Ophthalmology and Visual Science Case Western Reserve University Cleveland Ohio USA; ^7^ Division of Stem Cell Transplantation and Cellular Therapies, Department of Internal Medicine II University Hospital Schleswig‐Holstein Kiel, Kiel University Kiel Germany; ^8^ Faculty of Medicine Carl Gustav Carus TUD Dresden University of Technology Dresden Germany

**Keywords:** aging, epigenetic, hematopoietic, hsc, human, parabiosis, transplantation

## Abstract

Allogenic hematopoietic stem cell transplantation is a therapeutic procedure performed over a wide range of donor and recipient age combinations, representing natural experiments of how the age of the recipient affects aging in transplanted donor cells in vivo. We measured DNA methylation and epigenetic aging in donors and recipients and found that biological epigenetic clocks are accelerated in cells transplanted into an older body and decelerated in a younger body. This is the first evidence that the age of the circulating environment influences human epigenetic aging in vivo.

## Graphical abstract

Epigenetic aging is accellerated in cells living in an older body compared to cells living in a younger body.

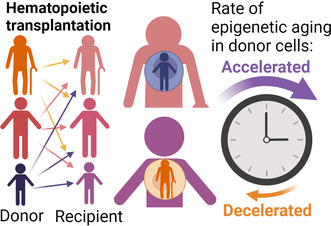

Hematopoietic stem cell transplantation introduces donor stem cells to a recipient with the intent of expanding to a full donor‐derived immune system. Matched sibling donors are preferred when available, but many transplantations are performed from unrelated donors. For unrelated donors, age is the most influential factor for long‐term survival after hematopoietic transplantation with 2‐year survival being 3% higher with a 10 years younger donor (Shaw et al., 2018). In rodents, connecting the circulatory system of a young and an older individual (heterochronic parabiosis) famously improves the physiology and extends lifespan of the older animal (Conboy et al., 2005; Zhang et al., 2023) while accelerating deterioration in the younger animal (Villeda et al., 2011). Single‐cell studies of different organs after heterochronic parabiosis have demonstrated that rodent hematopoietic stem cells are strongly affected by changes in the circulating environment (Ma et al., 2022), suggesting that also human hematopoietic cells may benefit from a rejuvenated circulating environment.

Here, we exploit the natural experiment of stem cell transplantation between differently aged individuals to study if the rate of intrinsic epigenetic aging in human immune cells is affected by differences in age between donor cells and the recipient body. We integrated two previously collected DNA methylation datasets from a German (Stölzel et al., 2017) and a Norwegian (Søraas et al., 2019) cohort of allogeneic hematopoietic transplantations and expanded the Norwegian cohort considerably with new donor and recipient pairs as well as longer follow‐up measurements of the existing pairs. The goal was to collect several longitudinal measurements from donor–recipient pairs with wide age differences. The cohorts and data analysis pipelines are described in detail in the Supporting Information Methods section. For hypothesis testing, mixed linear models were applied for each epigenetic clock, with chronological age, imputed immune cell proportions, sample batch and time after transplantation as fixed effects, and the donor–recipient‐pair‐identifier as random effect (Model 1). Ten epigenetic clocks were chosen—four trained to predict chronological epigenetic age (PCHannum [Higgins‐Chen et al., 2022], based on the Hannum clock [Hannum et al., 2013], Horvath2013 [Horvath, 2013], PCHorvath2013 [Higgins‐Chen et al., 2022], and PCSkinBlood [Higgins‐Chen et al., 2022] based on the Skin&Blood/Horvath2 clock [Horvath et al., 2018]), five trained to predict disease‐ and mortality‐linked biological measurements (PCPhenoAge [Higgins‐Chen et al., 2022], based on DNAmPhenoAge [Levine et al., 2018], GrimAge [Lu, Quach, et al., 2019], PCGrimAge [Higgins‐Chen et al., 2022], GrimAge2 [Lu et al., 2022], and DunePACE [Belsky et al., 2020]), and one to estimate the telomere lengths from DNA methylation (PCDNAmTL [Higgins‐Chen et al., 2022; Lu, Seeboth, et al., 2019]). We first compared the relative change in epigenetic aging between donor and recipient measurements for transplantations into a relatively younger or older recipient. Epigenetic aging acceleration was significantly different between the two groups for all biological epigenetic clocks (+4.1 median years of accelerated GrimAge2 in an older recipient, −1.3 median years of acceleration in a younger recipient, *p*.adj = 1.9e^−6^) as well as the epigenetic telomere length estimate, but not the chronological epigenetic clocks (Figure 1a).

**FIGURE 1:**
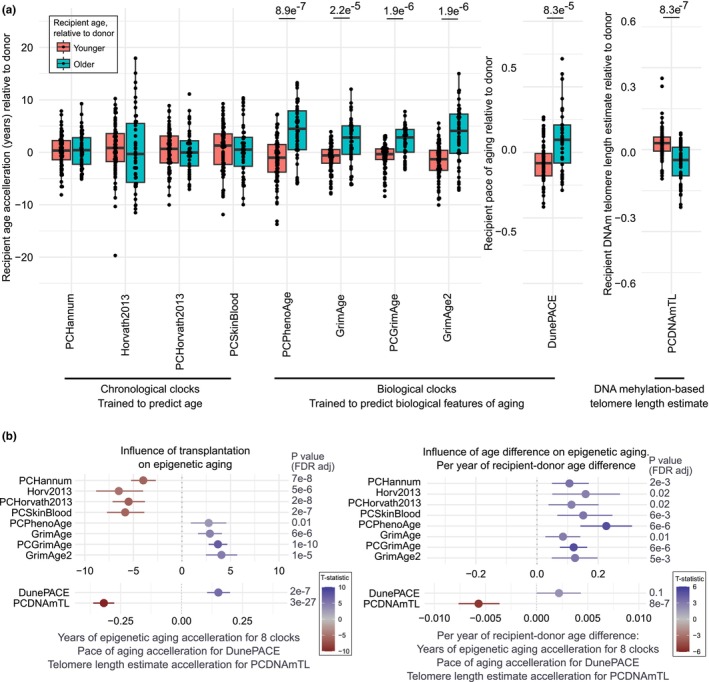
The effect of hematopoietic transplantation and recipient–donor age difference on epigenetic aging. (a) Linear mixed models with chronological age, imputed immune cells proportions, sample batch, years after transplantation, and donor–recipient status as fixed effects and donor–recipient‐pair as random effect (Model 1) were run for each epigenetic clock. The difference between recipient and donor residuals was compared for transplants into younger or older recipients. Boxplots shows the median, first and third quantile hinges and whiskers extended to 1.5 * IQR of the hinges. *p*‐values are from a Mann–Whitney *U* test, FDR‐adjusted for multiple testing. *p*‐values of comparisons not printed are >0.05. (b) Visualization of two variables from the linear mixed models. Influence of transplantation refers to the categorical variable DR [R] annotating samples to be donor or recipient. Influence of recipient–donor age difference on recipient measurements refers to the interaction variable DR [R] * recipDonDiff which has the recipient–donor age difference for the recipient measurements. The x‐axis shows the model estimates of epigenetic aging measurements with 95% confidence intervals. Full details of all variables in the models are included in Supporting Information: Tables S1 and S2.

We next interrogated the mixed models to study the influence of transplantation itself and the linear influence of recipient–donor age difference (in contrast to the categorical younger–older comparison in Figure 1a) on the rate of epigenetic aging in recipient measurements. Transplantation itself causes significantly increased biological epigenetic aging (4.07 years [95% CI = 2.38–5.76] for GrimAge2, *p*.adj = 1.4e^−5^) and the recipient–donor age difference causes significantly increased rate of biological epigenetic aging in recipient samples (0.12 years [95% CI = 0.05–0.20] for GrimAge2 per year of recipient–donor difference, *p*.adj = 5.2e^−3^). While chronological epigenetic clock estimates were not significantly different between younger and older recipients in the categorical comparison (Figure 1a), when considering a linear influence of the recipient–donor age difference (Figure 1b), also these clocks are influenced by the age difference with an acceleration when transplanted into an older body and deceleration when transplanted into a younger recipient. Interestingly, we find the influence of transplantation itself is directionally opposite for chronological clocks compared to biological clocks—hematopoietic transplantation and immune system expansion simultaneously decelerates chronological clocks and accelerate biological clocks. The DNAm telomere length estimator (PCDNAmTL) showed a strong effect of transplantation itself, as expected from published direct measurements of telomere lengths after hematopoietic transplantation (Lee et al., 1999). Interestingly, the recipient–donor age difference effect also had a significant influence on PCDNAmTL measurements. We highlight that this is a DNAm‐based estimation of telomere length, and we cannot exclude that this predicted telomeric change may be confounded by broad epigenetic rejuvenation. Direct measurement of telomere lengths in donors and recipients for a wide range of recipient–host age differences will be required to establish if this is true telomeric rejuvenation. The full details of the linear mixed models of all 10 investigated epigenetic clocks are in Supporting Information: Tables S1 and S2.

By plotting the residuals of the mixed model for a representative biological epigenetic clock (GrimAge2), we can see the relative trends between the donor and recipient samples relative to the recipient–donor age difference (Figure 2a). Another way to look at this data is to subtract the donor measurement from the corresponding recipient measurement to highlight the relative change in age acceleration due to being in a different body (Figure 2b, other clocks in Figure S3). In general, there is an enrichment of recipient deceleration at the left side (older cells transplanted to a younger host) and acceleration at the right side (younger cells transplanted to an older host), but there is also considerable variance. Finally, we investigated how the epigenetic aging measurements developed over time for transplantations into younger or older recipients by calculating the relative aging acceleration of recipient relative to the corresponding donor (Figure 2c). The divergence between transplantations into younger or older recipients is at its largest, ~1 year after transplantation. Several biological aging clocks appear to revert to the rate of epigenetic aging in the donor cells after the initial divergence due to the recipient–donor age difference. We do not have enough measurements to conclude strongly on the dynamics of these processes over time, but a stabilization of the rate of epigenetic aging would be consistent with the recently described dynamic reversibility of epigenetic aging changes due to cellular stress (Poganik et al., 2023).

**FIGURE 2:**
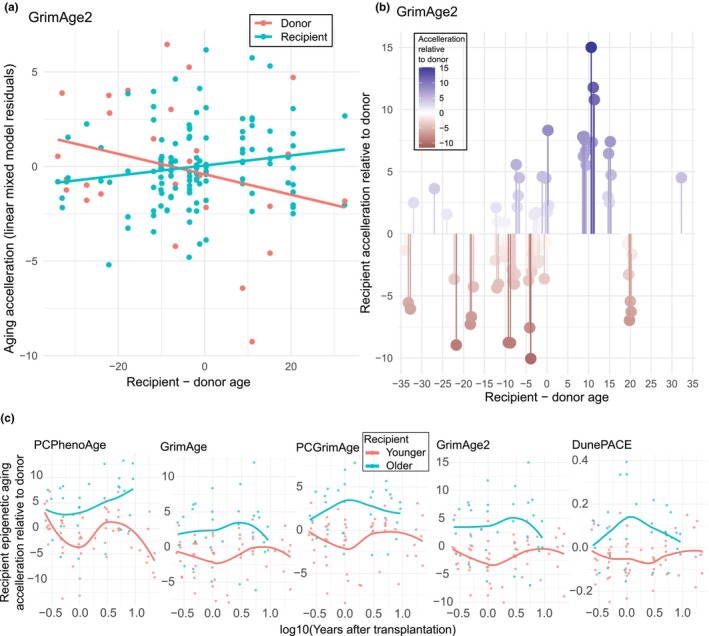
A detailed view of recipient–donor measurements for GrimAge2 and changes to epigenetic aging of biological clocks over time. (a) Linear mixed models with chronological age, imputed immune cells proportions, sample batch, years after transplantation, and donor–recipient status as fixed effects and donor–recipient‐pair as random effect (Model 1) were calculated and shown are all residuals for the GrimAge2 model. A linear regression is fitted to each set of donor and recipient measurements. (b) By subtracting the recipient residuals by the corresponding donor residuals, the relative change in epigenetic aging acceleration relative to the donor measurement is calculated. The same plot for other clocks is in Figure S3. (c) The recipient–donor residuals plotted against log10 (time after transplantation) for all five biological epigenetic clocks. Y‐axis shows years for PCPhenoAge, GrimAge, PCGrimAge, and GrimAge2 and pace of aging for DunePACE.

We hypothesize the following sequence of events to explain the observed epigenetic aging changes after transplantation into a younger or older host: First, engraftment and expansion of transplanted donor stem cells into a functioning immune system categorically decelerates chronological and accelerates biological epigenetic aging clocks through unknown mechanisms. Then, the age of the host environment influences the rate of epigenetic clock progression in the donor cells over time with a maximum divergence between younger and older environment around 1 year after transplantation. The most important implication of our findings is to establish that human cells in vivo are influenced by the age of their environment, giving support to accelerate human trials to dilute and/or rejuvenate an aging host environment.

## AUTHOR CONTRIBUTIONS

A.S., P.H., and J.A.D. conceived the study. P.H. performed data analysis, interpretation, and wrote the draft manuscript. A.S., M.I., M.M.A., T.G., J.B., M.W., F.S., and S.M. contacted and collected samples from donors and patients. S.H.B., S.H., and J.A.D. contributed to statistical analysis and interpretation. All authors gave input on the draft manuscript and approved its submission.

## FUNDING INFORMATION

This work was made possible by funding from the The Norwegian Cancer Association, The Research Council of Norway, and The South Eastern Norway Health Authority.

## CONFLICT OF INTEREST STATEMENT

Steve Horvath is a founder and paid consultant of the nonprofit Epigenetic Clock Development Foundation that licenses patents related to several epigenetic clocks. Arne Søraas owns shares in Age Labs, a company working on predicting disease based on epigenetic data. No Age Labs technology or algorithms were used in this study.

## Supporting information


Data S1.

Appendix S1.

## Data Availability

The DNA methylation array measurements that were used to calculate epigenetic aging clocks along with donor and recipient ages and time since transplantation have been deposited to Mendeley Data, doi 10.17632/j5krtjfj6y.1.

## References

[acel14112-bib-0001] Belsky, D. W. , Caspi, A. , Arseneault, L. , Baccarelli, A. , Corcoran, D. , Gao, X. , Hannon, E. , Harrington, H. L. , Rasmussen, L. J. H. , Houts, R. , Huffman, K. , Kraus, W. E. , Kwon, D. , Mill, J. , Pieper, C. F. , Prinz, J. , Poulton, R. , Schwartz, J. , Sugden, K. , … Moffitt, T. E. (2020). Quantification of the pace of biological aging in humans through a blood test, the DunedinPoAm DNA methylation algorithm. eLife, 9, 1–56. 10.7554/eLife.54870 PMC7282814 32367804

[acel14112-bib-0002] Conboy, I. M. , Conboy, M. J. , Wagers, A. J. , Girma, E. R. , Weismann, I. L. , & Rando, T. A. (2005). Rejuvenation of aged progenitor cells by exposure to a young systemic environment. Nature, 433(7027), 760–764. 10.1038/nature03260 15716955

[acel14112-bib-0003] Hannum, G. , Guinney, J. , Zhao, L. , Zhang, L. , Hughes, G. , Sadda, S. V. , Klotzle, B. , Bibikova, M. , Fan, J. B. , Gao, Y. , Deconde, R. , Chen, M. , Rajapakse, I. , Friend, S. , Ideker, T. , & Zhang, K. (2013). Genome‐wide methylation profiles reveal quantitative views of human aging rates. Molecular Cell, 49(2), 359–367. 10.1016/j.molcel.2012.10.016 23177740 PMC3780611

[acel14112-bib-0004] Higgins‐Chen, A. T. , Thrush, K. L. , Wang, Y. , Minteer, C. J. , Kuo, P.‐L. , Wang, M. , Niimi, P. , Sturm, G. , Lin, J. , Moore, A. Z. , Bandinelli, S. , Vinkers, C. H. , Vermetten, E. , Rutten, B. P. F. , Geuze, E. , Okhuijsen‐Pfeifer, C. , van der Horst, M. Z. , Schreiter, S. , Gutwinski, S. , … Levine, M. E. (2022). A computational solution for bolstering reliability of epigenetic clocks: Implications for clinical trials and longitudinal tracking. Nature Aging, 2(7), 644–661. 10.1038/s43587-022-00248-2 36277076 PMC9586209

[acel14112-bib-0005] Horvath, S. (2013). DNA methylation age of human tissues and cell types. Genome Biology, 14, R115. 24138928 10.1186/gb-2013-14-10-r115 PMC4015143

[acel14112-bib-0006] Horvath, S. , Oshima, J. , Martin, G. M. , Lu, A. T. , Quach, A. , Cohen, H. , Felton, S. , Matsuyama, M. , Lowe, D. , Kabacik, S. , Wilson, J. G. , Reiner, A. P. , Maierhofer, A. , Flunkert, J. , Aviv, A. , Hou, L. , Baccarelli, A. A. , Li, Y. , Stewart, J. D. , … Raj, K. (2018). Epigenetic clock for skin and blood cells applied to Hutchinson Gilford Progeria Syndrome and ex vivo studies. Aging, 10(7), 1758–1775. 10.18632/aging.101508 30048243 PMC6075434

[acel14112-bib-0007] Lee, J. J. , Kook, H. , Chung, I. J. , Kim, H. J. , Park, M. R. , Kim, C. J. , Nah, J. A. , & Hwang, T. J. (1999). Telomere length changes in patients undergoing hematopoietic stem cell transplantation. Bone Marrow Transplantation, 24(4), 411–415. 10.1038/sj.bmt.1701923 10467331

[acel14112-bib-0008] Levine, M. E. , Lu, A. T. , Quach, A. , Chen, B. H. , Assimes, T. L. , Bandinelli, S. , Hou, L. , Baccarelli, A. A. , Stewart, J. D. , Li, Y. , Whitsel, E. A. , Wilson, J. G. , Reiner, A. P. , Aviv, A. , Lohman, K. , Liu, Y. , Ferrucci, L. , & Horvath, S. (2018). An epigenetic biomarker of aging for lifespan and healthspan. Aging, 10(4), 573–591. 10.18632/aging.101414 29676998 PMC5940111

[acel14112-bib-0009] Lu, A. T. , Binder, A. M. , Zhang, J. , Yan, Q. , Reiner, A. P. , Cox, S. R. , Corley, J. , Harris, S. E. , Kuo, P.‐L. , Moore, A. Z. , Bandinelli, S. , Stewart, J. D. , Wang, C. , Hamlat, E. J. , Epel, E. S. , Schwartz, J. D. , Whitsel, E. A. , Correa, A. , Ferrucci, L. , … Horvath, S. (2022). DNA methylation GrimAge version 2. Aging, 14(23), 9484–9549. 10.18632/aging.204434 36516495 PMC9792204

[acel14112-bib-0010] Lu, A. T. , Quach, A. , Wilson, J. G. , Reiner, A. P. , Aviv, A. , Raj, K. , Hou, L. , Baccarelli, A. A. , Li, Y. , Stewart, J. D. , Whitsel, E. A. , Assimes, T. L. , Ferrucci, L. , & Horvath, S. (2019). DNA methylation GrimAge strongly predicts lifespan and healthspan. Aging, 11(2), 303–327. 10.18632/aging.101684 30669119 PMC6366976

[acel14112-bib-0011] Lu, A. T. , Seeboth, A. , Tsai, P. C. , Sun, D. , Quach, A. , Reiner, A. P. , Kooperberg, C. , Ferrucci, L. , Hou, L. , Baccarelli, A. A. , Li, Y. , Harris, S. E. , Corley, J. , Taylor, A. , Deary, I. J. , Stewart, J. D. , Whitsel, E. A. , Assimes, T. L. , Chen, W. , … Horvath, S. (2019). DNA methylation‐based estimator of telomere length. Aging, 11(16), 5895–5923. 10.18632/aging.102173 31422385 PMC6738410

[acel14112-bib-0012] Ma, S. , Wang, S. , Ye, Y. , Ren, J. , Chen, R. , Li, W. , Li, J. , Zhao, L. , Zhao, Q. , Sun, G. , Jing, Y. , Zuo, Y. , Xiong, M. , Yang, Y. , Wang, Q. , Lei, J. , Sun, S. , Long, X. , Song, M. , … Liu, G.‐H. (2022). Heterochronic parabiosis induces stem cell revitalization and systemic rejuvenation across aged tissues. Cell Stem Cell, 29(6), 990–1005.e10. 10.1016/j.stem.2022.04.017 35613617

[acel14112-bib-0013] Poganik, J. R. , Zhang, B. , Baht, G. S. , Tyshkovskiy, A. , Deik, A. , Kerepesi, C. , Yim, S. H. , Lu, A. T. , Haghani, A. , Gong, T. , Hedman, A. M. , Andolf, E. , Pershagen, G. , Almqvist, C. , Clish, C. B. , Horvath, S. , White, J. P. , & Gladyshev, V. N. (2023). Biological age is increased by stress and restored upon recovery. Cell Metabolism, 35(5), 807–820.e5. 10.1016/j.cmet.2023.03.015 37086720 PMC11055493

[acel14112-bib-0014] Shaw, B. E. , Logan, B. R. , Spellman, S. R. , Marsh, S. G. E. , Robinson, J. , Pidala, J. , Hurley, C. , Barker, J. , Maiers, M. , Dehn, J. , Wang, H. , Haagenson, M. , Porter, D. , Petersdorf, E. W. , Woolfrey, A. , Horowitz, M. M. , Verneris, M. , Hsu, K. C. , Fleischhauer, K. , & Lee, S. J. (2018). Development of an unrelated donor selection score predictive of survival after HCT: Donor age matters most. Biology of Blood and Marrow Transplantation, 24(5), 1049–1056. 10.1016/j.bbmt.2018.02.006 29454040 PMC5953795

[acel14112-bib-0015] Søraas, A. , Matsuyama, M. , de Lima, M. , Wald, D. , Buechner, J. , Gedde‐Dahl, T. , Søraas, C. L. , Chen, B. , Ferrucci, L. , Dahl, J. A. , Horvath, S. , & Matsuyama, S. (2019). Epigenetic age is a cell‐intrinsic property in transplanted human hematopoietic cells. Aging Cell, 18(2), e12897. 10.1111/acel.12897 30712319 PMC6413751

[acel14112-bib-0016] Stölzel, F. , Brosch, M. , Horvath, S. , Kramer, M. , Thiede, C. , Von Bonin, M. , Ammerpohl, O. , Middeke, M. , Schetelig, J. , Ehninger, G. , Hampe, J. , & Bornhäuser, M. (2017). Dynamics of epigenetic age following hematopoietic stem cell transplantation. Haematologica, 102(8), e321–e323. 10.3324/HAEMATOL.2016.160481 28550187 PMC5541887

[acel14112-bib-0017] Villeda, S. A. , Luo, J. , Mosher, K. I. , Zou, B. , Britschgi, M. , Bieri, G. , Stan, T. M. , Fainberg, N. , Ding, Z. , Eggel, A. , Lucin, K. M. , Czirr, E. , Park, J. S. , Couillard‐Després, S. , Aigner, L. , Li, G. , Peskind, E. R. , Kaye, J. A. , Quinn, J. F. , … Wyss‐Coray, T. (2011). The ageing systemic milieu negatively regulates neurogenesis and cognitive function. Nature, 477(7362), 90–96. 10.1038/nature10357 21886162 PMC3170097

[acel14112-bib-0018] Zhang, B. , Lee, D. E. , Trapp, A. , Tyshkovskiy, A. , Lu, A. T. , Bareja, A. , Kerepesi, C. , McKay, L. K. , Shindyapina, A. V. , Dmitriev, S. E. , Baht, G. S. , Horvath, S. , Gladyshev, V. N. , & White, J. P. (2023). Multi‐omic rejuvenation and life span extension on exposure to youthful circulation. Nature Aging, 3, 948–964. 10.1038/s43587-023-00451-9 37500973 PMC11095548

