# Supplementary material for: Epigenetic aging of human blood cells is influenced by the age of the host body
Source: Aging Cell. 2024 Mar 4;23(5):e14112. doi: 10.1111/acel.14112 (PMC11113269; doi:10.1111/acel.14112)
Supplement: Supplementary file 1 — Data S1. [file ACEL-23-e14112-s001.docx]

**Supporting Information Methods**

**Cohorts**

The German cohort was previously described in detail ^1^. Briefly, it is a group of *de novo* AML patients and closely matched donors, transplanted with allogeneic peripheral blood stem cells, with complete donor chimerism, no AML relapse, and no donor-lymphocyte infusions performed. Donor samples were collected at the time of transplantation and recipients measured multiple times over several years. Samples were measured in two batches of DNA methylation arrays (Illumina 450k) with some samples measured in both batches and others in only one batch. Thirteen donor-recipient pairs had both donor and recipient samples, in total 110 measurements. In addition, 10 recipients had 67 samples without corresponding donor samples.

The Norwegian cohort is based on a previously described cohort of allogeneic peripheral blood hematopoietic transplant donors and recipients ^2^ that was expanded considerably for this present study. The goal was to obtain several measurements over time of donor-recipient pairs with wide age differences. The previously published measurements of this cohort were from five donor-recipient pairs, with 12 measurements of donors and recipients performed on the Illumina 450k platform. For this study, six additional donor-recipient pairs were recruited and 26 measurements were collected from this new group over the months and years after transplantation. We also collected five additional longer follow-up measurements of the previously published five donor-recipient pairs. In addition to donor-recipient pairs, we collected a set of independent recipient measurements, 50 unique recipients with 72 total samples. All the new measurements for this present study were performed on the Illumina 850K EPIC platform. The diagnoses of the 11 patients in the Norwegian cohort are as follows: 4 *de novo* AML, 1 AML-therapy-induced-MDS, 2 ALL, 1 beta-thalassemia, 1 NHL, 1 SCID. One patient in the Norwegian cohort consented to participating in our study but opted out of medical history sharing. All donors and recipients provided written consent to study participation. The study was approved by the Regional Committee for Research Ethics in Norway (REK approvals #13412 and #19468).

**DNA methylation measurements**

Peripheral blood collected in EDTA tubes were snap frozen on dry ice and stored at -80°C until DNA extraction. Samples collected as dried blood spots were collected from participants using Whatman (LIPIDX) collection cards. DNA methylation was measured on Illumina EPIC arrays by Life and Brain GmBH, Bonn, Germany.

**Raw data processing**

All raw data was processed starting from .idat files with R for this analysis. Based on a recent study that compared different DNA methylation raw data processing pipelines for epigenetic clocks ^3^ we decided on using ENmix ^4^ with the suggested optimal parameters for epigenetic clocks from the comparison: Out-of-bag background correction, RELIC dye-bias correction, quantile normalization separately for methylated and unmethylated intensities and RCP correction of probe design type bias. The folder with raw .idat files was first loaded with ENmix::readidat() then profiled by ENmix:qcinfo() with default settings. Loaded data was then sequentially applied to ENmix::preprocessENmix(), ENmix::norm.quantile() and ENmix::rcp() with the above described parameters. ENmix::qcfilter() was then applied, removing very low quality CpGs and samples detected by the qcinfo() function and imputing missing values. The result from this pipeline is a table of DNA methylation beta values. This pipeline was run separately for each batch of measurements.

**Immune cell imputation**

For immune cell imputation we leaned on a comparison of different algorithms ^5^ and chose the robust partial correlations (RPC) with a conservative core of six abundant immune cell populations that could be imputed in all samples with relatively high confidence. The epidish R package was used ^6^ through the epidish::epidish() function with the contained centDHSbloodDMC.m[1:6] reference set of CpGs for imputation.

**Epigenetic clocks**

Eight epigenetic clocks were chosen for this study: Hannum ^7^, Horvath2013 ^8^, SkinBlood ^9^, DNAmTL ^10^, PhenoAge ^11^, GrimAge ^12^, GrimAge2 ^13^, DunePACE ^14^. A computational trick based on principal components to reduce noise and variance of many of these clocks was recently published ^15^ and we used only this newer version for the Hannum (PCHannum), SkinBlood (PCSkinBlood), PhenoAge (PCPhenoAge) and DNAmTL (PCDNAmTL). For the Horvath2013 and GrimAge clocks we studied both the original and the PC-based versions: PCHorvath2013 and PCGrimAge. The PC-based clocks and DunePACE were calculated using R code published to github while the Horvath2013 clock coefficients were derived from the original publication. GrimAge and GrimAge2 were calculated with R scripts locally. All epigenetic clock calculations were performed inside our secure environment to handle patient data in a GDPR-compliant manner.

**Recipient samples from < 0.1 years after transplantation**

When comparing imputed immune cell composition as well as epigenetic clocks for samples collected at different times after transplantation, we noticed a strong divergence of samples collected less than 0.1 years after transplantation (Supporting Information Figure 1 a, c, f). Because hematopoietic transplantation involves re-establishing a full immune system from transplanted stem cells, we reasoned that the observed divergence before 0.1 years after transplantation was likely due to strong changes in the population early on with epigenetic changes which are different from the data that both the immune cell imputation and epigenetic clocks were trained on. To avoid any uncertainty or bias, we decided to exclude recipient samples < 0.1 years after transplantation from further analysis, representing 10 samples in total. In Supporting Information Figure 1 b, d, g we show the dataset after excluding these 10 samples.

**Time after transplantation bias. Statistical adjustment**

Hematopoietic transplantation has previously been described to cause a predictable set of dynamic changes in epigenetic aging measured by the Horvath2013 clock in the months and years after transplantation ^1^. We confirm this dynamic also in our data and we see similar patterns in additional clocks: PCHorvath2013, PCSkinBlood and to a lesser extent PCPhenoAge (Supporting Information Figure 1 d, g). Other epigenetic clocks (PCHannum, GrimAge, GrimAge2, PCGrimAge, DunePACE, PCDNAmTL) show no systematic trends in epigenetic aging changes in the time after transplantation. We found that the time after transplantation bias appeared linear or log-linear (Supporting Information Figure 1 d, g). When statistically adjusting for the time after transplantation, this bias was mostly corrected for the affected clocks (Supporting Information Figure 1 e, h). In Supporting Information Figure 2 we include a sensitivity analysis related to this adjustment for the time after transplantation bias. Model 1 linearly adjusts for time after transplantation and this is the main statistical model used throughout this paper. Model 2 adjusts for log10(time after transplantation) while Model 3 does not have any adjustment for time after transplantation. The epigenetic clocks that showed a time after transplantation bias generally showed both a stronger influence of transplantation and a stronger influence of the age difference after adjusting for the time after transplantation (compare Model 1 to Model 3). The linear adjustment appears to be very similar to log10-adjustment (compare Model 1 to Model 2) so we decided to use the linear adjustment to minimize complexity. It is important to note that the biological epigenetic clocks had little or no time after transplantation bias and for these clocks the statistical adjustment for time after transplantation was irrelevant for the interpretation of the statistical model details (compare Model 1,2,3 for PCPhenoAge, GrimAge, PCGrimAge, GrimAge2, DunePACE).

**Hypothesis testing. Linear mixed model details**

Donor and corresponding recipient measurements can be interpreted as a longitudinal series, following the same cells in two different individuals. Our data also contained several follow-up measurements of recipients over longer periods of time. Because of this longitudinal nature of the measurements and that different donor-recipient-pairs can have different numbers of measurements, mixed models were appropriate for hypothesis testing in this data. We applied linear mixed models in a similar way as linear regressions have previously been applied to define intrinsic epigenetic aging acceleration (IEAA) where the effect of sample chronological age as well as immune cell composition is adjusted for, representing a measurement of intrinsic cellular aging acceleration relative to expected aging. In addition to chronological age and immune cell composition, we also added a categorical variable with sample batch (to adjust for any batch-specific and/or illumina platform-specific effects) as the fixed effects and the categorical donor-recipient-pair identifier as random effect.

**Influence of clinical covariates: aGvHD, cGvHD, infections**

From a previous analysis of the german cohort DNA methylation data ^1^, it was known that the Horvath 2013 chronological clock is affected by chronic graft-versus-host-disease (cGvHD). To study the influence of cGvHD as well as acute graft-versus-host-disease (aGvHD) and severe infections on the epigenetic aging measurements we included aGvHD, cGvHD and infections as covariates in the linear mixed models (Supporting Information Figure 5 & 6, Model 4). Adjusting for these covariates had only minor effects on the biological epigenetic clocks, but several chronological clocks no longer had a significant effect from the recipient-donor age difference in Model 4 (Supporting Information Figure 5). The affected chronological clocks were Horvath2013, PCHorvath1 and PCHorvath2, supporting previous findings showing the Horvath2013 clock being affected by cGvHD ^1^. When interrogating the separate effects from the covariates introduced in Model 4, we find that these three chronological clocks are significantly affected by all these covariates (aGvHD, cGvHD and infections), but with the most significant influence from cGvHD. Notably, PCHannum and PCDNAmTL were also significantly affected by cGvHD. Importantly, none of the biological epigenetic clocks were significantly influenced by these three covariates.

**Recipient measurements without a donor reference**

Most of the analysis presented in this manuscript exploits the donor measurement as a baseline reference to look for the relative change in recipient epigenetic aging after transplantation. Because obtaining matched donor and recipient samples is challenging, we also collected a set of recipient measurements with wide recipient-donor age difference where we do not have a reference donor measurement. Without a donor reference, we do not see any statistically significant difference in aging acceleration between transplants into a younger or older recipient (Supporting Information Figure 4). Having a donor reference allows a longitudinal analysis that removes variation between individuals that is a well-known weakness of epigenetic aging measurements at the population level. This analysis highlights the importance of longitudinal measurements of epigenetic aging before and after the intervention to track the effect of the intervention.

**References**

1. Stölzel, F. *et al.* Dynamics of epigenetic age following hematopoietic stem cell transplantation. *Haematologica* **102**, e321–e323 (2017).

2. Søraas, A. *et al.* Epigenetic age is a cell-intrinsic property in transplanted human hematopoietic cells. *Aging Cell* **18**, e12897 (2019).

3. Ori, A. P. S., Lu, A. T., Horvath, S. & Ophoff, R. A. Significant variation in the performance of DNA methylation predictors across data preprocessing and normalization strategies. *Genome Biology* **23**, (2022).

4. Xu, Z., Niu, L., Li, L. & Taylor, J. A. ENmix: a novel background correction method for Illumina HumanMethylation450 BeadChip. *Nucleic Acids Res* **44**, e20 (2016).

5. Teschendorff, A. E., Breeze, C. E., Zheng, S. C. & Beck, S. A comparison of reference-based algorithms for correcting cell-type heterogeneity in Epigenome-Wide Association Studies. *BMC Bioinformatics* **18**, 105 (2017).

6. Zheng, S. C. *et al.* A novel cell-type deconvolution algorithm reveals substantial contamination by immune cells in saliva, buccal and cervix. *Epigenomics* **10**, 925–940 (2018).

7. Hannum, G. *et al.* Genome-wide Methylation Profiles Reveal Quantitative Views of Human Aging Rates. *Molecular Cell* **49**, 359–367 (2013).

8. Horvath, S. DNA methylation age of human tissues and cell types. *Genome Biology* (2013).

9. Horvath, S. *et al.* Epigenetic clock for skin and blood cells applied to Hutchinson Gilford Progeria Syndrome and ex vivo studies. *Aging* **10**, 1758–1775 (2018).

10. Lu, A. T. *et al.* GWAS of epigenetic aging rates in blood reveals a critical role for TERT. *Nature Communications* **9**, 1–13 (2018).

11. Levine, M. E. *et al.* An epigenetic biomarker of aging for lifespan and healthspan. *Aging* **10**, 573–591 (2018).

12. Lu, A. T. *et al.* DNA methylation GrimAge strongly predicts lifespan and healthspan. *Aging* **11**, 303–327 (2019).

13. Lu, A. T. *et al.* DNA methylation GrimAge version 2. *Aging* **14**, 9484–9549 (2022).

14. Belsky, D. W. *et al.* Quantification of the pace of biological aging in humans through a blood test, the DunedinPoAm DNA methylation algorithm. *eLife* **9**, 1–56 (2020).

15. Higgins-Chen, A. T. *et al.* A computational solution for bolstering reliability of epigenetic clocks: implications for clinical trials and longitudinal tracking. *Nat Aging* **2**, 644–661 (2022).
