# Supplementary material for: Epigenetic aging of human blood cells is influenced by the age of the host body
Source: Aging Cell. 2024 Mar 4;23(5):e14112. doi: 10.1111/acel.14112 (PMC11113269; doi:10.1111/acel.14112)
Supplement: Supplementary file 2 — Appendix S1. [file ACEL-23-e14112-s002.pdf]

Supporting Information Figure 1

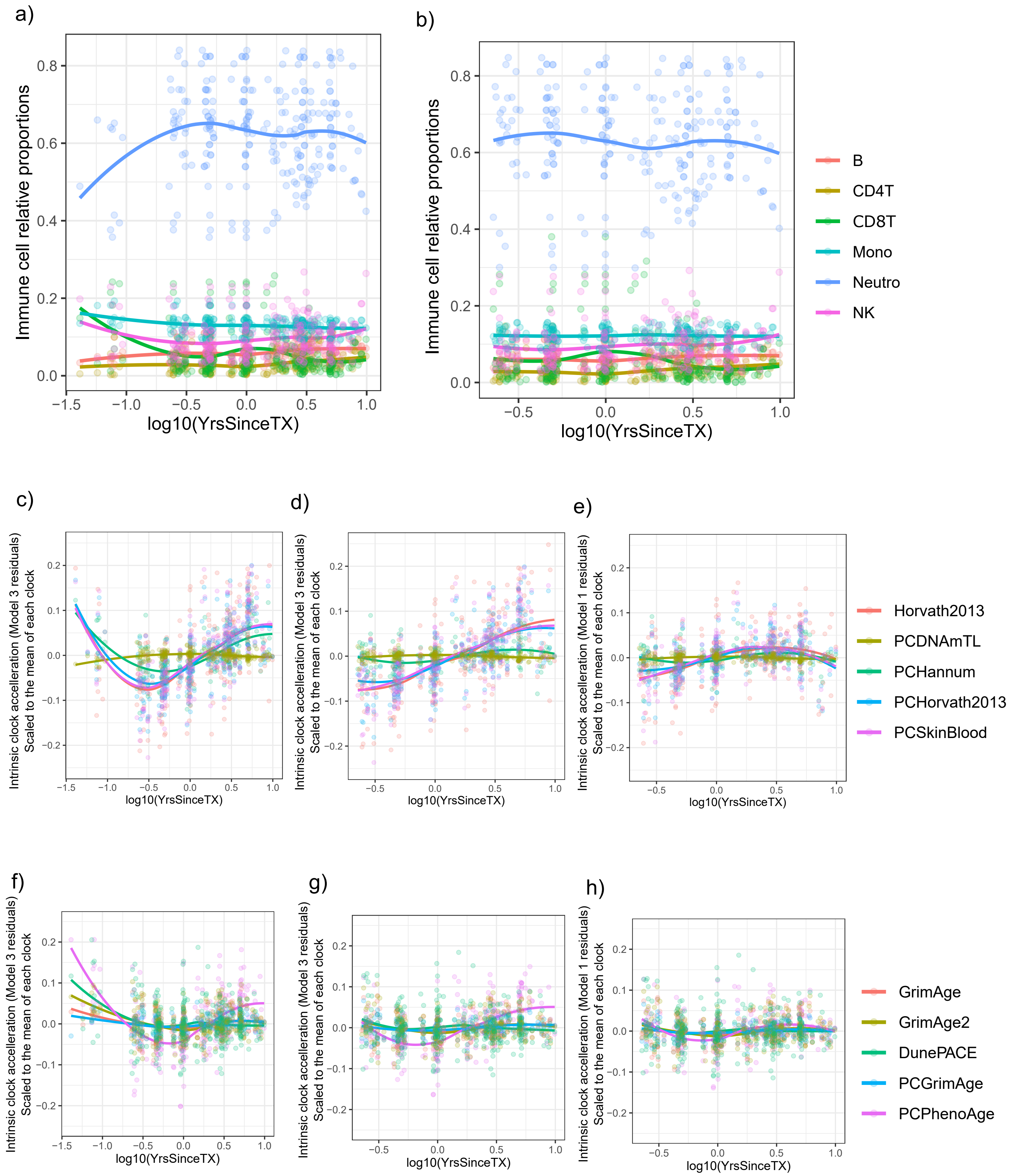

Supporting Information Figure 2

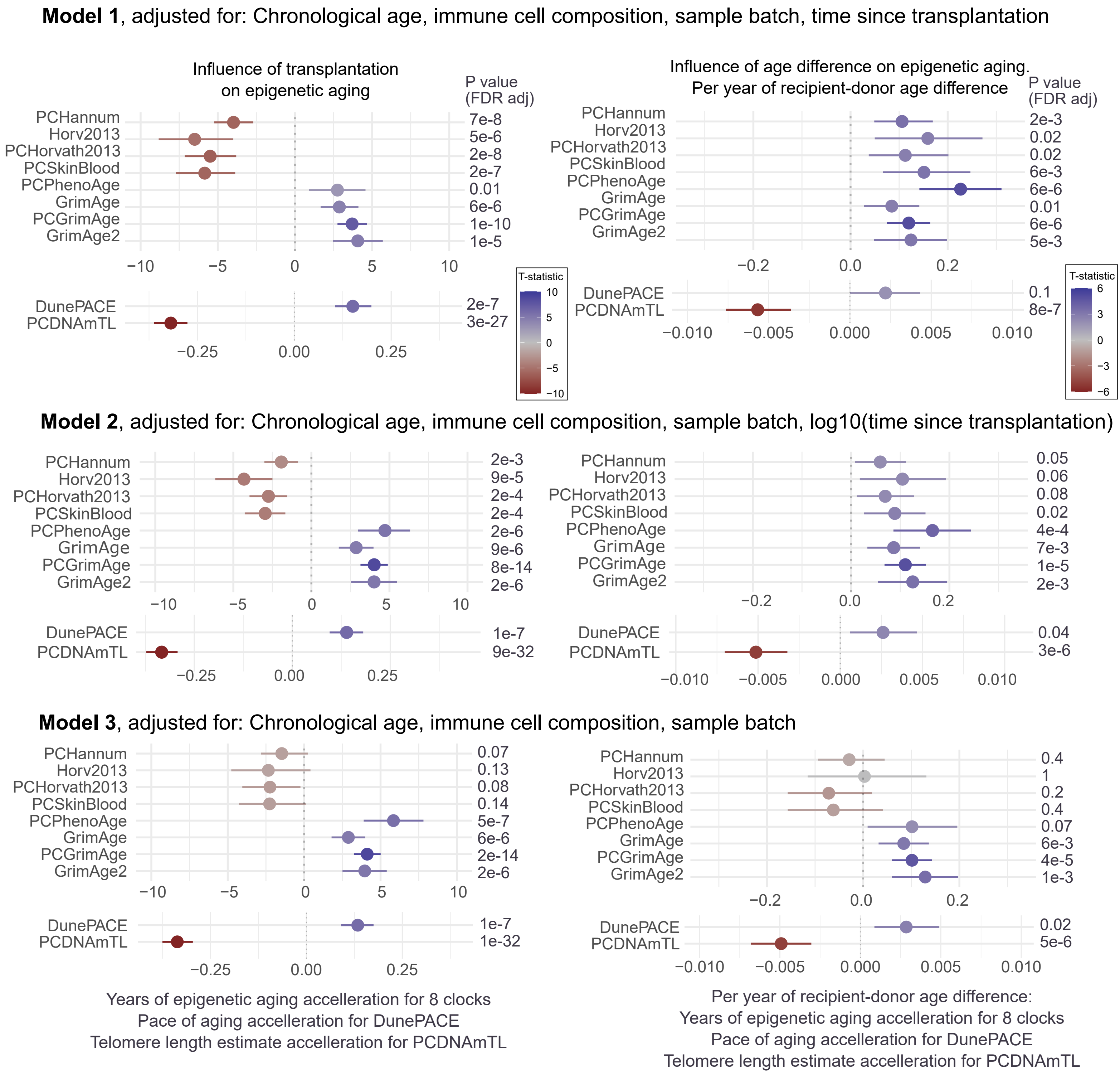

Supporting Information Figure 3

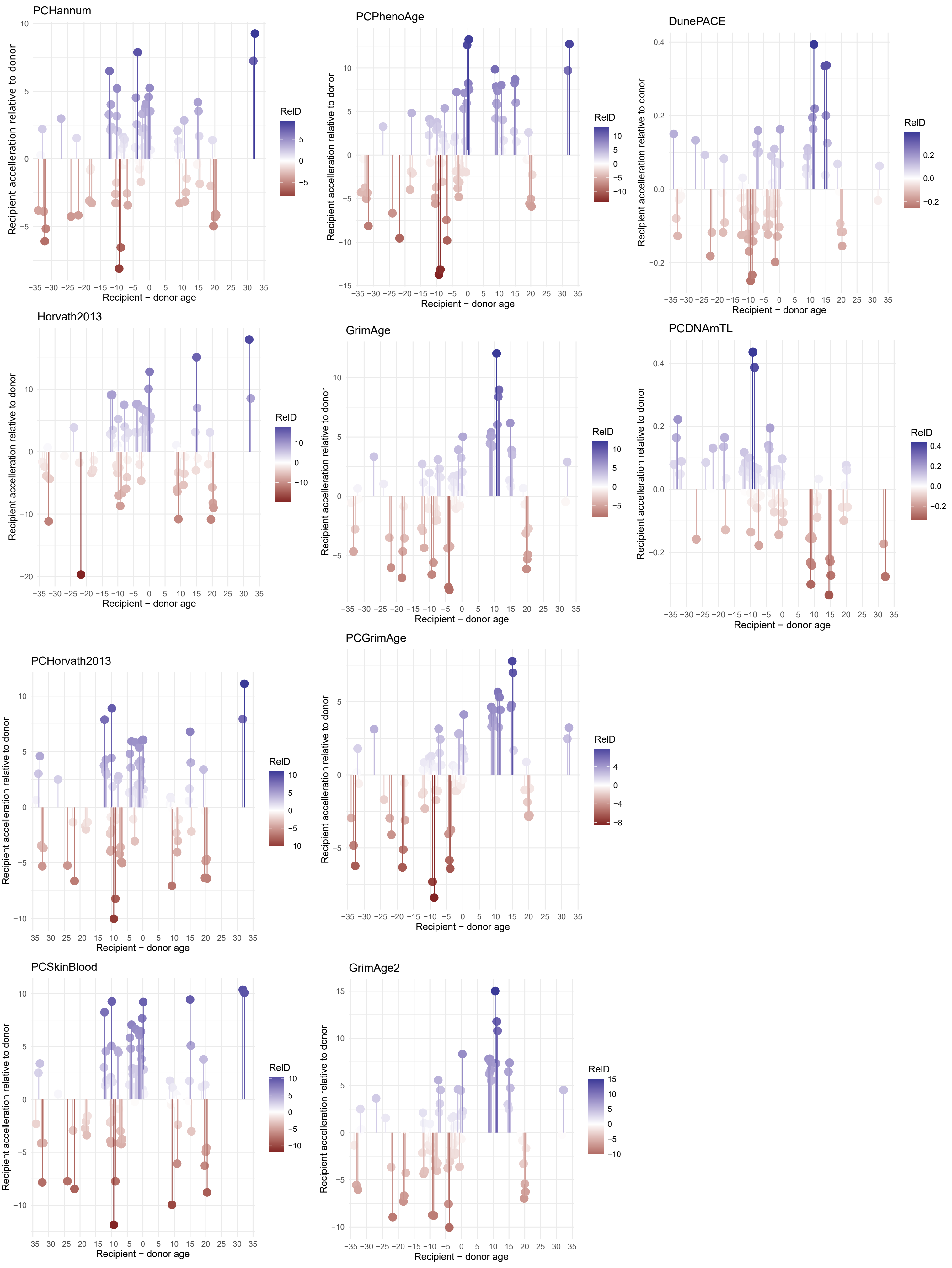

Supporting Information Figure 4

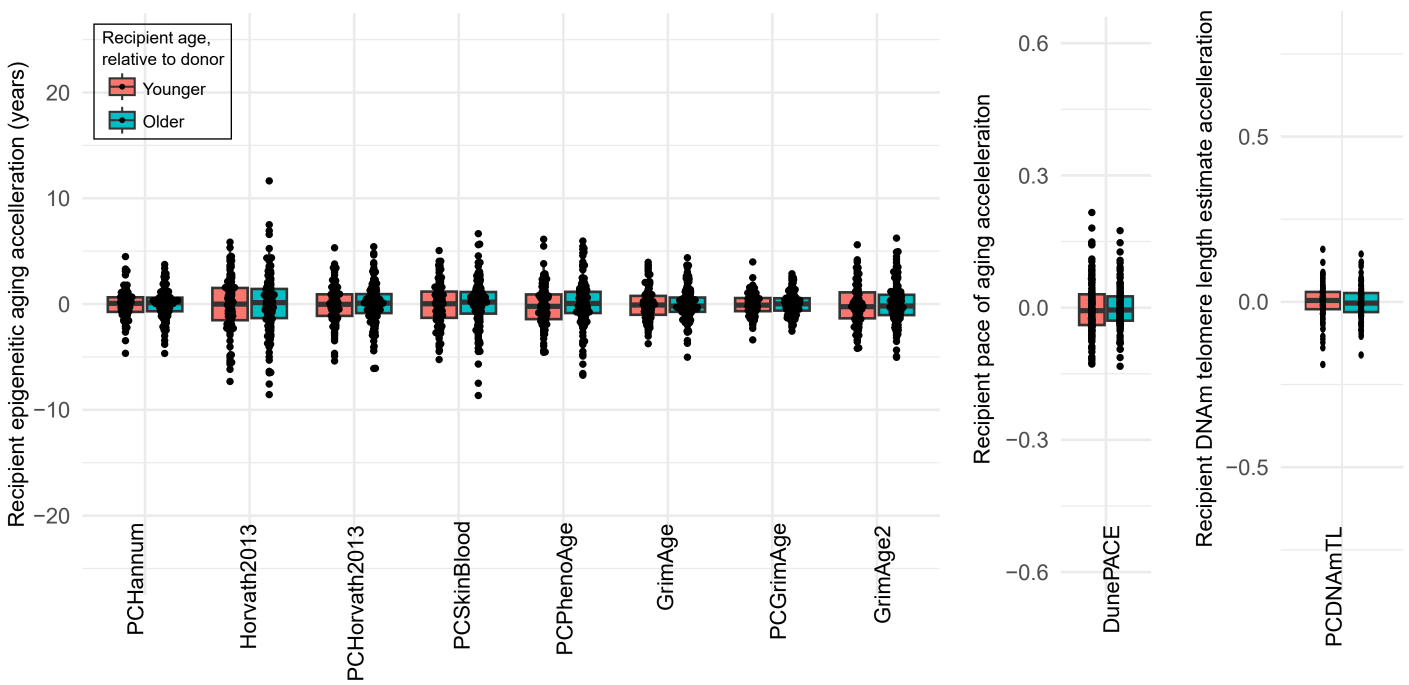

Supporting Information Figure 5

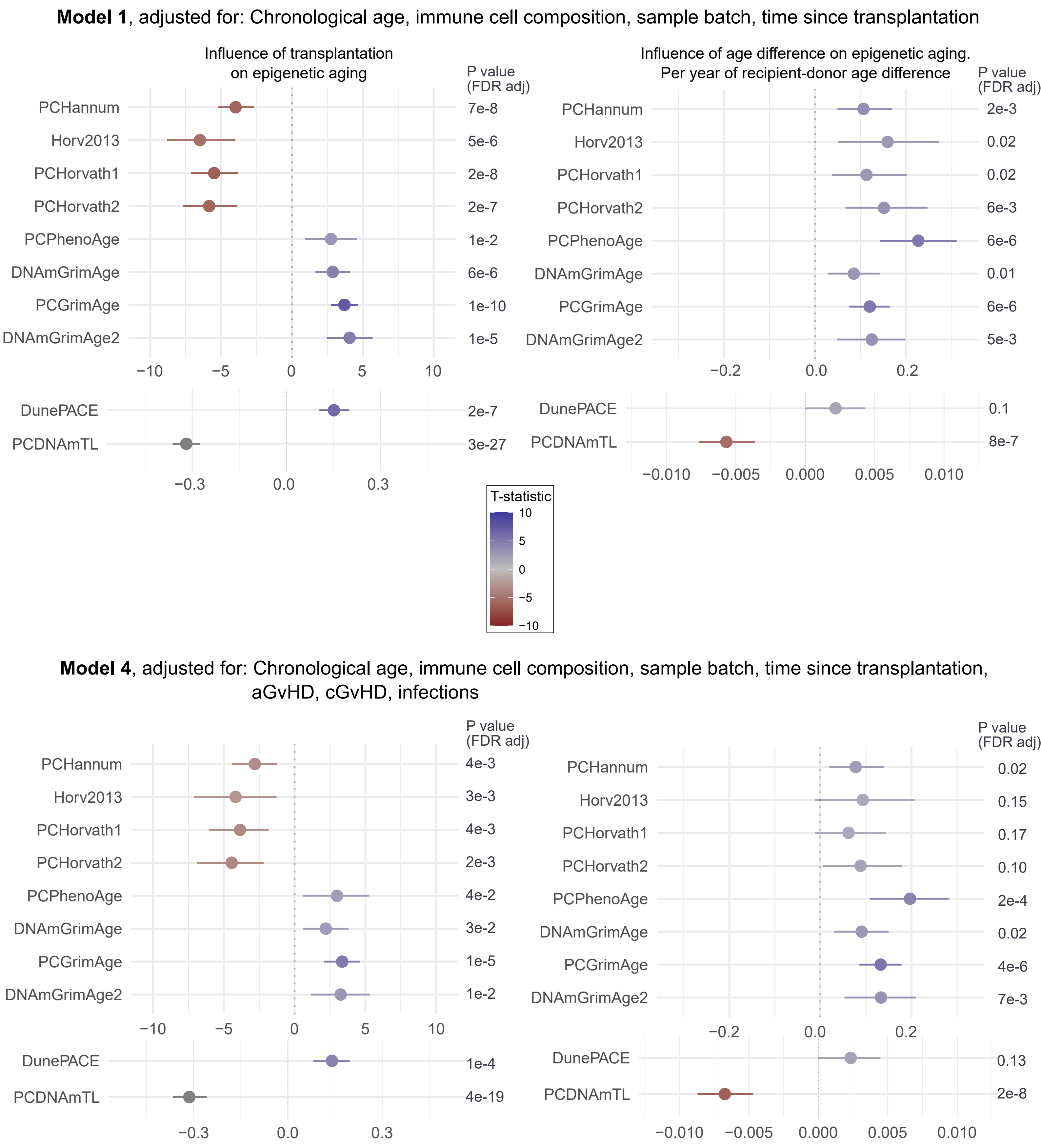

Supporting Information Figure 6

Model 4, adjusted for: Chronological age, immune cell composition, sample batch, time since transplantation, aGvHD, cGvHD, infections

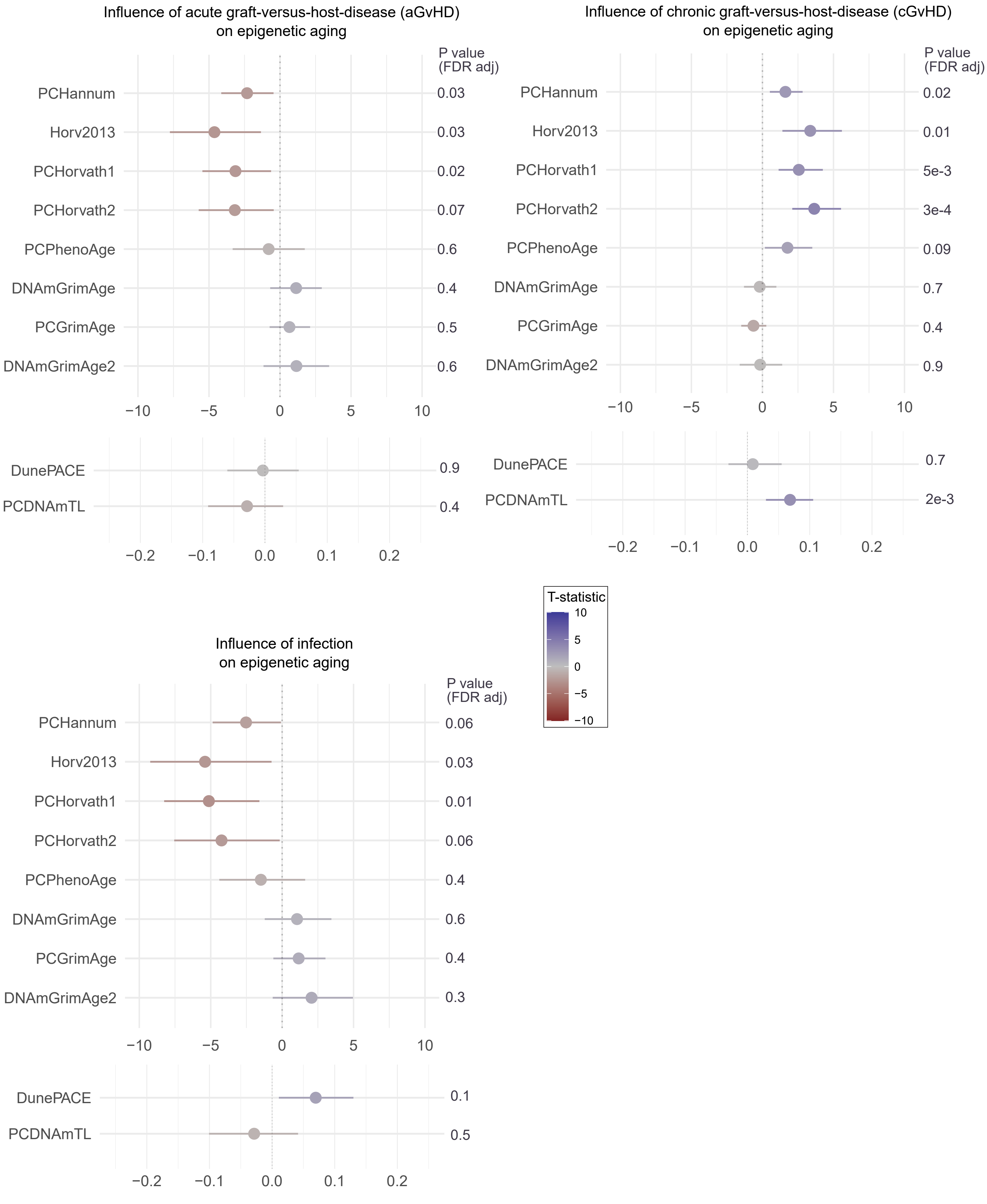

Supporting Information Table 1

	PCHannum			Horv2013			PCHorvath1			PCHorvath2			PCDNAmTL		
Predictors	*Estimates*	*Statistic*	*p*	*Estimates*	*Statistic*	*p*	*Estimates*	*Statistic*	*p*	*Estimates*	*Statistic*	*p*	*Estimates*	*Statistic*	*p*
(Intercept)	-7.94 (-20.76 – 4.88)	-1.22	2.229e-01	-27.93 (-50.64 – -5.23)	-2.43	**3.055e-02**	-34.92 (-52.40 – -17.45)	-3.95	**4.646e-04**	-21.84 (-40.63 – -3.05)	-2.30	6.917e-02	6.88 (6.47 – 7.30)	33.06	**1.051e-65**
DR [R]	-3.98 (-5.29 – -2.66)	-5.99	**6.795e-08**	-6.50 (-9.00 – -4.00)	-5.13	**4.799e-06**	-5.49 (-7.21 – -3.77)	-6.31	**1.870e-08**	-5.84 (-7.82 – -3.87)	-5.85	**1.763e-07**	-0.32 (-0.36 – -0.27)	-13.96	**3.635e-27**
AgeCells	1.24 (1.07 – 1.41)	14.58	**2.065e-28**	1.43 (1.21 – 1.65)	13.02	**1.707e-24**	1.59 (1.34 – 1.83)	12.67	**1.327e-23**	1.27 (1.04 – 1.49)	10.95	**3.106e-19**	-0.02 (-0.02 – -0.02)	-9.32	**1.379e-15**
immuRPC6 B	29.97 (10.42 – 49.52)	3.03	**4.370e-03**	52.91 (15.86 – 89.95)	2.82	**1.634e-02**	29.28 (3.59 – 54.96)	2.25	**4.297e-02**	16.94 (-12.39 – 46.27)	1.14	3.482e-01	2.17 (1.50 – 2.83)	6.41	**8.266e-09**
immuRPC6 CD4T	-20.69 (-41.22 – -0.17)	-1.99	5.554e-02	9.02 (-29.80 – 47.85)	0.46	6.465e-01	-4.43 (-31.36 – 22.50)	-0.33	7.454e-01	-11.90 (-42.70 – 18.90)	-0.76	5.217e-01	1.95 (1.25 – 2.65)	5.49	**5.653e-07**
immuRPC6 CD8T	-16.67 (-34.56 – 1.22)	-1.84	7.239e-02	26.68 (-6.78 – 60.13)	1.58	1.514e-01	22.97 (-0.58 – 46.52)	1.93	7.615e-02	28.84 (2.06 – 55.62)	2.13	8.750e-02	0.66 (0.05 – 1.26)	2.16	**4.487e-02**
immuRPC6 Mono	24.43 (6.61 – 42.26)	2.71	**1.035e-02**	30.10 (-3.32 – 63.53)	1.78	1.157e-01	25.23 (1.74 – 48.73)	2.12	5.320e-02	26.00 (-0.68 – 52.67)	1.93	9.335e-02	1.19 (0.58 – 1.79)	3.89	**2.956e-04**
immuRPC6 Neutro	17.63 (7.02 – 28.25)	3.28	**2.435e-03**	19.13 (-0.95 – 39.22)	1.88	1.029e-01	18.65 (4.71 – 32.59)	2.64	**1.957e-02**	16.15 (0.22 – 32.07)	2.01	9.042e-02	0.89 (0.53 – 1.25)	4.85	**7.143e-06**
YrsAfterReceivingTX	0.69 (0.55 – 0.83)	9.83	**1.096e-16**	0.91 (0.66 – 1.16)	7.16	**3.479e-10**	0.93 (0.74 – 1.11)	9.93	**6.110e-17**	1.06 (0.85 – 1.26)	10.18	**1.444e-17**	-0.00 (-0.01 – 0.00)	-1.86	8.220e-02
Batch [set1]	-10.23 (-13.58 – -6.87)	-6.03	**6.795e-08**	-10.02 (-16.05 – -4.00)	-3.29	**4.785e-03**	-5.53 (-9.98 – -1.08)	-2.46	**2.859e-02**	-1.82 (-6.79 – 3.16)	-0.72	5.217e-01	0.03 (-0.08 – 0.14)	0.60	5.868e-01
Batch [set9]	-7.63 (-10.41 – -4.85)	-5.42	**7.826e-07**	-2.60 (-7.56 – 2.36)	-1.04	3.235e-01	0.73 (-2.99 – 4.44)	0.39	7.454e-01	3.88 (-0.24 – 7.99)	1.86	9.684e-02	0.13 (0.04 – 0.22)	2.85	**8.531e-03**
Batch [Stoz1]	-8.61 (-14.09 – -3.12)	-3.10	**3.891e-03**	-4.10 (-11.15 – 2.95)	-1.15	2.912e-01	-2.68 (-11.31 – 5.94)	-0.62	6.226e-01	0.16 (-7.10 – 7.42)	0.04	9.655e-01	0.11 (-0.03 – 0.24)	1.59	1.313e-01
Batch [Stoz2]	-11.69 (-17.16 – -6.22)	-4.23	**1.087e-04**	-9.31 (-16.31 – -2.31)	-2.63	**2.041e-02**	-6.29 (-14.90 – 2.33)	-1.44	1.890e-01	-2.55 (-9.78 – 4.68)	-0.70	5.217e-01	0.17 (0.04 – 0.30)	2.63	**1.436e-02**
recipDonDiff	0.17 (0.01 – 0.34)	2.06	5.189e-02	0.16 (-0.04 – 0.37)	1.56	1.514e-01	0.36 (0.10 – 0.63)	2.73	**1.795e-02**	0.22 (0.00 – 0.44)	1.99	9.042e-02	0.00 (-0.00 – 0.00)	0.31	7.605e-01
DR [R] * recipDonDiff	0.11 (0.04 – 0.17)	3.40	**1.889e-03**	0.16 (0.04 – 0.27)	2.72	**1.867e-02**	0.11 (0.03 – 0.19)	2.74	**1.795e-02**	0.15 (0.06 – 0.24)	3.24	**5.688e-03**	-0.01 (-0.01 – -0.00)	-5.37	**8.343e-07**
**Random Effects**															
σ^2	3.92			14.63			6.68			8.91			0.00		
τ_{00}	32.52 $_{Pair}$			36.87 $_{Pair}$			87.75 $_{Pair}$			52.27 $_{Pair}$			0.01 $_{Pair}$		
ICC	0.89			0.72			0.93			0.85			0.74		
N	24 $_{Pair}$			24 $_{Pair}$			24 $_{Pair}$			24 $_{Pair}$			24 $_{Pair}$		
Observations	153			153			153			153			153		
Marginal R^2 / Conditional R^2	0.876 / 0.987			0.878 / 0.965			0.805 / 0.986			0.813 / 0.973			0.814 / 0.952		

Supporting Information Table 2

	PCPhenoAge			DNAmGrimAge			PCGrimAge			DNAmGrimAge2			DunePACE		
Predictors	*Estimates*	*Statistic*	*p*	*Estimates*	*Statistic*	*p*	*Estimates*	*Statistic*	*p*	*Estimates*	*Statistic*	*p*	*Estimates*	*Statistic*	*p*
(Intercept)	-15.80 (-33.18 – 1.59)	-1.80	1.018e-01	4.70 (-7.40 – 16.80)	0.77	5.550e-01	7.72 (-1.78 – 17.21)	1.61	2.079e-01	3.47 (-12.07 – 19.02)	0.44	8.242e-01	1.28 (0.85 – 1.72)	5.84	**2.792e-07**
DR [R]	2.76 (0.85 – 4.67)	2.85	**1.081e-02**	2.88 (1.60 – 4.16)	4.44	**5.540e-05**	3.71 (2.71 – 4.71)	7.32	**1.434e-10**	4.07 (2.38 – 5.76)	4.77	**1.403e-05**	0.15 (0.10 – 0.20)	6.08	**1.723e-07**
AgeCells	1.25 (1.08 – 1.42)	14.51	**3.195e-28**	1.04 (0.90 – 1.18)	14.38	**6.456e-28**	1.00 (0.89 – 1.12)	17.29	**5.627e-35**	0.97 (0.81 – 1.14)	11.74	**1.566e-21**	0.00 (0.00 – 0.01)	2.91	**1.275e-02**
immuRPC6 B	-35.26 (-63.52 – -7.00)	-2.47	**2.786e-02**	-23.41 (-42.43 – -4.38)	-2.43	**3.049e-02**	-17.97 (-32.81 – -3.12)	-2.39	5.424e-02	-34.72 (-59.70 – -9.73)	-2.75	**1.461e-02**	-0.47 (-1.20 – 0.25)	-1.28	3.020e-01
immuRPC6 CD4T	-21.89 (-51.53 – 7.74)	-1.46	1.829e-01	-12.48 (-32.46 – 7.50)	-1.24	3.282e-01	0.88 (-14.72 – 16.47)	0.11	9.116e-01	-17.86 (-44.08 – 8.37)	-1.35	2.705e-01	-1.34 (-2.09 – -0.58)	-3.49	**3.298e-03**
immuRPC6 CD8T	-26.34 (-51.90 – -0.78)	-2.04	6.524e-02	-21.77 (-39.12 – -4.42)	-2.48	**3.049e-02**	-5.12 (-18.67 – 8.44)	-0.75	6.228e-01	-28.52 (-51.20 – -5.84)	-2.49	**2.352e-02**	-1.04 (-1.68 – -0.39)	-3.17	**7.054e-03**
immuRPC6 Mono	5.76 (-19.76 – 31.29)	0.45	6.559e-01	-14.15 (-31.43 – 3.13)	-1.62	1.796e-01	-3.57 (-17.06 – 9.93)	-0.52	7.528e-01	-13.32 (-35.94 – 9.30)	-1.16	3.357e-01	-0.72 (-1.37 – -0.07)	-2.18	7.794e-02
immuRPC6 Neutro	22.09 (6.77 – 37.42)	2.85	**1.081e-02**	-2.52 (-12.85 – 7.80)	-0.48	6.859e-01	12.58 (4.52 – 20.65)	3.09	**9.196e-03**	0.70 (-12.86 – 14.25)	0.10	9.851e-01	-0.37 (-0.76 – 0.03)	-1.84	1.269e-01
YrsAfterReceivingTX	0.70 (0.51 – 0.90)	7.25	**2.124e-10**	0.00 (-0.13 – 0.14)	0.05	9.594e-01	0.10 (-0.00 – 0.20)	1.92	1.420e-01	-0.02 (-0.20 – 0.15)	-0.29	8.951e-01	-0.00 (-0.01 – 0.00)	-1.64	1.727e-01
Batch [set1]	-9.82 (-14.44 – -5.20)	-4.20	**1.792e-04**	13.55 (10.33 – 16.76)	8.34	**5.532e-13**	-2.04 (-4.56 – 0.47)	-1.60	2.079e-01	25.08 (20.93 – 29.22)	11.98	**7.750e-22**	0.02 (-0.09 – 0.14)	0.41	7.337e-01
Batch [set9]	-1.89 (-5.69 – 1.91)	-0.98	3.779e-01	10.61 (7.95 – 13.26)	7.90	**4.103e-12**	-0.81 (-2.89 – 1.27)	-0.77	6.228e-01	16.02 (12.61 – 19.43)	9.29	**1.277e-15**	-0.04 (-0.13 – 0.06)	-0.79	5.876e-01
Batch [Stoz1]	-6.05 (-11.55 – -0.55)	-2.18	5.226e-02	16.14 (11.62 – 20.67)	7.06	**2.944e-10**	-0.47 (-4.10 – 3.17)	-0.25	8.563e-01	25.61 (20.39 – 30.82)	9.72	**1.418e-16**	0.02 (-0.10 – 0.14)	0.30	7.665e-01
Batch [Stoz2]	-8.63 (-14.10 – -3.16)	-3.12	**6.584e-03**	1.85 (-2.66 – 6.35)	0.81	5.550e-01	-0.72 (-4.34 – 2.90)	-0.39	8.004e-01	6.87 (1.69 – 12.06)	2.62	**1.825e-02**	-0.03 (-0.15 – 0.08)	-0.56	6.959e-01
recipDonDiff	0.08 (-0.09 – 0.24)	0.91	3.881e-01	0.03 (-0.10 – 0.17)	0.47	6.859e-01	0.06 (-0.06 – 0.17)	0.99	5.400e-01	-0.00 (-0.16 – 0.15)	-0.01	9.915e-01	-0.00 (-0.00 – 0.00)	-0.52	6.959e-01
DR [R] * recipDonDiff	0.23 (0.14 – 0.31)	5.07	**6.446e-06**	0.08 (0.03 – 0.14)	2.81	**1.438e-02**	0.12 (0.07 – 0.17)	5.07	**6.272e-06**	0.12 (0.05 – 0.20)	3.14	**5.171e-03**	0.00 (-0.00 – 0.00)	1.91	1.238e-01
**Random Effects**															
σ^2	8.49			3.76			2.29			6.58			0.01		
τ_{00}	23.22 $_{Pair}$			19.64 $_{Pair}$			13.00 $_{Pair}$			22.80 $_{Pair}$			0.01 $_{Pair}$		
ICC	0.73			0.84			0.85			0.78			0.57		
N	24 $_{Pair}$			24 $_{Pair}$			24 $_{Pair}$			24 $_{Pair}$			24 $_{Pair}$		
Observations	153			153			153			153			153		
Marginal R^2 / Conditional R^2	0.890 / 0.971			0.896 / 0.983			0.909 / 0.986			0.884 / 0.974			0.461 / 0.766		

Supporting Information Table 3

	German cohort		Norwegian cohort	
	Recipient younger than donor	Recipient older than donor	Recipient younger than donor	Recipient older than donor
Number of donor-recipient pairs	9	4	8	3
Mean age donor at time of transplantation (95%CI)	45.8 (35.5 - 56.1)	33.2 (24.7 - 41.8)	41.1 (29.8 - 52.4)	28.7 (10.4 - 47)
Mean age recipient at time of transplantation (95%CI)	38.3 (28 - 48.6)	43.4 (21.7 - 65.1)	17.6 (3.23 - 32)	51 (33.1 - 68.8)
Female % donors	22.2%	0	37.5%	0
Female % recipients	22.2%	0	37.5%	0
Total measurements	70	40	29	14
Number of follow-up measurements per donor-recipient pair after transplantation (95%CI)	6.78 (4.86 - 8.69)	9 (6.75 - 11.3)	2.12 (1.3 - 2.95)	3.33 (-0.461 - 7.13)
Mean time of measurements after transplantation (years, 95%CI)	1.87 (1.39 - 2.35)	2.31 (1.93 - 2.7)	11.7 (4.86 - 18.4)	5.92 (-5.19 - 17)
